# Prevalence and determinants of chronic kidney disease among community-dwelling adults, 50 years and older in Ireland

**DOI:** 10.1093/ckj/sfaf065

**Published:** 2025-03-11

**Authors:** Meera Tandan, Leonard D Browne, Amir Jalali, Colm Rowan, Frank Moriarty, Austin G Stack

**Affiliations:** School of Medicine, University of Limerick, Limerick, Ireland; School of Medicine, University of Limerick, Limerick, Ireland; Health Research Institute (HRI), University of Limerick, Limerick, Ireland; School of Medicine, University of Limerick, Limerick, Ireland; Department of Nephrology, University Hospital Limerick, Limerick, Ireland; School of Pharmacy and Biomolecular Science, Royal College of Surgeon in Ireland, Dublin, Ireland; School of Medicine, University of Limerick, Limerick, Ireland; Health Research Institute (HRI), University of Limerick, Limerick, Ireland; Department of Nephrology, University Hospital Limerick, Limerick, Ireland

**Keywords:** chronic kidney disease, comorbid condition, prevalence, risk factor, social deprivation

## Abstract

**Background:**

Using the Irish Longitudinal Study on Ageing (TILDA), we evaluated the prevalence and distribution of chronic kidney disease (CKD), and its determinants in order to identify risk groups for population health planning in Ireland.

**Methods:**

Data were analysed from Wave 1 (2009–2011) of the TILDA, a national cohort of participants aged 50+ years who had both plasma creatinine and cystatin C measured at baseline. Kidney function was estimated using the 2012 and 2021 Chronic Kidney Disease Epidemiology Collaboration (CKD-EPI) equations. CKD was defined as estimated glomerular filtration rate <60 mL/min/1.73 m^2^. Multivariable logistic regression explored associations using adjusted odds ratios (OR).

**Results:**

Prevalence of CKD was significantly higher using the CKD-EPI 2012_(Scr-CysC)_ compared with the CKD-EPI 2021_(Scr-CysC)_ (14.7% vs 11.3%, respectively). The prevalence was highest in patients with cardiovascular disease (CVD) (33.9%), diabetes (28.0%), cancer (25.5%), urinary incontinence (23.7%), bone diseases (21.5%), hypertension (19.8%) and obesity (19.5%). In multivariable analysis, individuals with hypertension (OR 1.78), diabetes (OR 1.45), CVD (OR 1.43), cancer (OR 1.53), overweight (OR 1.37) and obesity (OR 2.33) experienced greater likelihood of CKD. In addition, individuals with a history of previous hospitalization (OR 1.50), free or subsidized healthcare (OR 1.31), and unemployed individuals (OR 1.86) were also significantly more likely to have CKD.

**Conclusion:**

Compared with the national average, the burden of CKD is far greater in older individuals with major chronic conditions and socioeconomic deprivation. The identification and targeting of these groups through national surveillance programmes is likely to yield substantial benefits from more effective disease management and proactive population health planning.

## Graphical Abstract

**Graphical Abstract:**
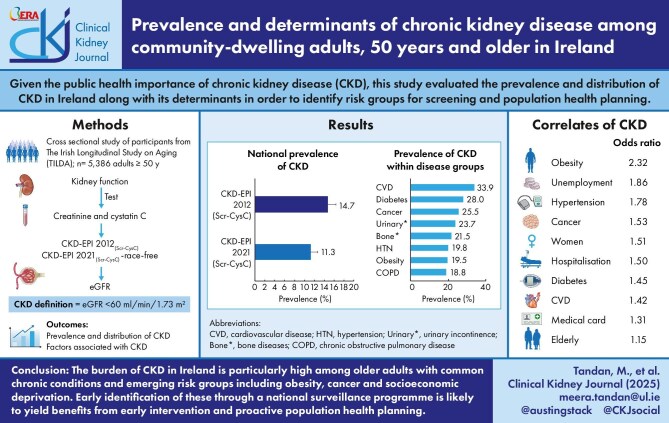

KEY LEARNING POINTS **What was known:**

- Prevalence of chronic kidney disease (CKD) is high in Ireland, particularly among older adults.
- Traditional risk factors such as diabetes and hypertension are principal drivers of rising CKD burden.

**This study adds:**

- Prevalence of CKD was far higher than the national average among several emerging risk groups including cancer, chronic obstructive pulmonary disease, bone disease and obesity.
- Cancer, obesity and several socioeconomic indicators contribute to CKD burden independent of traditional risk factors.
- The choice of estimated glomerular filtration rate (eGFR) equation influences the estimate of CKD burden in a population with significantly lower values for Chronic Kidney Disease Epidemiology Collaboration (CKD-EPI) 2021_(Scr-CysC)_ than for CKD-EPI 2012_(Scr-CysC)_ (11.3% vs 14.7%).

**Potential impact:**

- This national study highlights the large burden of CKD among traditional risk groups and emerging risk groups and emphasizes the importance of integrating social determinants of health into future prediction models for CKD prevalence.
- It draws attention to large variation in prevalence estimates that is derived from using different eGFR equations with important consequences for risk classification, healthcare planning and resource allocation.
- It also identifies high-risk groups that should be targeted for CKD screening through national surveillance programmes in order to maximize the benefits of early diagnosis and intervention.

## INTRODUCTION

Chronic kidney disease (CKD) is a major global health concern, ranked as the 8th leading cause of death, and contributes to 3.16 million deaths annually and significant healthcare costs [1–4]. Moreover, CKD substantially impacts disability-adjusted life years (DALYs), rising from 14th to 8th position since 1990 [1], and is projected to become the 5th leading cause of years lost by 2040 [4]. The high burden of CKD is driven by rising prevalence of traditional risk factors including diabetes, hypertension and obesity, an ageing population [5–7], and emerging socioeconomic factors that accelerate its occurrence and consequences [8].

Estimation of CKD burden and distribution of risk factors at the population level is crucial for disease management and prevention strategies [9–11]. CKD prevalence, influenced by both incidence and patient survival, is an important summary metric of disease occurrence, as it influences public health policy and clinical strategy. In Europe, CKD Stage 3–5 prevalence ranges from 1% in central Italy to 5.9% in Northeast Germany, a variation that is not fully explained by traditional risk factors [11]. In the USA, CKD Stage 3–5 prevalence varies from 4.8% in the Northeast to 11.8% in the Midwest regions [12]. The reasons for this heterogeneity are not fully understood. It is unclear whether these differences are due to differences in creatinine-based equations to estimate glomerular filtration rate (GFR) or differences in the distribution of underlying risk factors. Furthermore, it is uncertain whether and to what extent social determinants of health independently contribute to CKD prevalence beyond traditional CKD risk factors.

In Ireland, the prevalence of CKD among older individuals has been addressed in several studies. The 2007 Survey of Lifestyle, Attitudes and Nutrition (SLAN) found a prevalence of 11.6% (9.0%–14.2%) in adults >45 years [13]. The National Kidney Disease Surveillance System (NKDSS) reported a prevalence of 9.7% in adults aged 45 years and over, like SLAN, while more recently, the Irish Longitudinal Study on Ageing (TILDA), a nationally representative longitudinal study on ageing with individuals age 50 years and over, reported a prevalence of 11.7% [14, 15]. Although these studies have provided important information on the overall burden of disease, they have not explored in-depth the prevalence distribution in high-risk groups with common chronic diseases that could be targeted for increased surveillance, nor have they considered the relative contribution of emerging lifestyle and socioeconomic factors to CKD, which may require consideration in an overarching preventative strategy.

Because of the public health importance of this condition, we evaluated the prevalence of CKD and its determinants using data from Wave 1 of TILDA. This data set was used to test our priori hypotheses that (i) traditional CKD risk factors, and (ii) behavioural and socioeconomic factors are independently associated with CKD. The availability of data on a comprehensive list of demographics, social, clinical, behavioural and treatment factors for a large, randomly selected, national sample of older individuals in Ireland provided us with a unique opportunity to investigate these relationships.

## MATERIALS AND METHODS

### Study design and participants

This cross-sectional study utilized data from the first wave of TILDA, a population-based prospective cohort study, representative of the community dwelling adults in Ireland, age 50 years and over. The design of TILDA is underpinned by a rigorous design and sampling frame [16]. The sampling frame was the Irish Geodirectory, a comprehensive and up-to-date listing and mapping of all residential addresses in the Republic of Ireland compiled by ‘An Post’ (the Irish Postal Service) and Ordnance Survey Ireland. The initial multi-stage probability sample of addresses was chosen by means of the RANSAM sampling procedure, developed by the Economic and Social Research Institute. TILDA ensured an unbiased participant selection through stratification by socio-economic group and geography and utilized a two-stage sampling process which ensured equal probability sampling, minimized bias and addressed non-responses with calibration weights. Data were captured on demographic characteristics, comorbid medical conditions, social and economic indicators, hospitalization and medication [16, 17]. From October 2009 to February 2011, 8175 participants completed a home-based, computer-assisted personal interview and participated in a comprehensive health assessment, carried out by trained nurses [18].

For this study we included participants aged 50 years and older, who had undergone an assessment of both plasma creatinine and cystatin C concentrations at baseline. Ethical approval was obtained from the Faculty of Health Sciences, Trinity College Dublin Research Ethics Committee, and informed consent was obtained from all participants. This study utilized anonymized dataset from TILDA that can be available through the Irish Social Science Data Archive at www.ucd.ie/issda [19].

### Measurement of plasma creatinine and cystatin C

During health assessments, trained nurses collected blood samples from individuals for measurement of creatinine and cystatin C [16]. Each sample was centrifuged and aliquoted into 10 bar-coded cryovials which were then stored at –80°C. Creatinine and cystatin C were measured simultaneously from frozen plasma [20]. Creatinine was measured using an enzymatic method traceable to isotope-dilution mass spectrometry (Roche Creatinine plus ver.2, Roche Diagnostics, Basel, Switzerland). Cystatin C was measured using a second-generation particle enhanced immunoturbidimetric assay (Roche Tina-quant™) on a Roche Cobas 701 analyser traceable to the European reference standard material (ERM-DA471/IFCC) for cystatin C [20].

### Measurement of kidney function and classification of CKD

The Chronic Kidney Disease Epidemiology Collaboration Consortium (CKD-EPI) equations 2012 and 2021 (race-free) were used to estimate eGFR using plasma creatinine and cystatin C in combination [21, 22]. For the primary analysis, participants with eGFR <60 mL/min/1.73 m^2^ were classified as having CKD. In addition, we examined the impact of an age-adapted definition of CKD to account for physiological aging of the kidney. For this we defined CKD using age-specific eGFR thresholds of <75 mL/min/1.73 m^2^ for individuals <40 years, <60 mL/min/1.73 m^2^ for those aged 40–65 years and <45 mL/min/1.73 m^2^ for individuals >65 years [23].

### Explanatory variables

Data were collected on demographic characteristics (age, sex), socioeconomic and behavioural factors (marital status, education, employment and smoking), financial support [individuals on free or subsidized healthcare by way of a medical card or general practirioner (GP) visit card], health service utilization (recent hospital admissions in past 12 months), comorbid medical conditions and prescribed medications. Data were collected from the computer-assisted personal interviews (CAPI). Comorbid conditions were based on participants’ self-reported physician-diagnosed conditions, confirmed by information from the CAPI questionnaire and medication labels. Diabetes was defined as self-reported physician-diagnosed diabetes, receipt of insulin or analogues, glucose-lowering medications, and those with an HbA1C level of ≥48 mmol/L. Cardiovascular disease (CVD) was classified as present if angina, myocardial infarction, congestive heart failure, cerebral vascular disease, transient ischaemic attack, angioplasty or stent placement was reported. Hypertension was defined as self-reported high blood pressure or mean systolic blood pressure (SBP) of ≥140 mmHg or mean diastolic blood pressure (DBP) ≥90 mmHg and/or on an antihypertensive medication. Chronic obstructive pulmonary disease (COPD) included bronchitis, emphysema and asthma, while bone disease encompassed arthritis (osteoarthritis or rheumatism) and osteoporosis. Mental health problems were defined as having emotional, nervous or psychiatric problems such as depression or anxiety and serious memory impairment. Cancer was defined by malignant tumours, leukaemia, lymphoma (excluding minor skin cancers) and prostate cancer. Anthropometric measurements were available for height and weight with determination of body mass index (BMI), and hip and waist circumference. Laboratory data on total cholesterol, high-density lipoprotein, low-density lipoprotein, triglycerides and HbA1C were also available.

### Statistical analysis

Descriptive statistics were used to calculate CKD prevalence, overall and in demographic and clinical subgroups using both CKD-EPI 2012_(Scr-CysC)_ and CKD-EPI 2021_(Scr-CysC)_ equations. Crude and weighted prevalence was calculated with survey weights adjusted for selection and non-response bias to the health assessment component of the survey. The population weights were calculated based on age, sex and educational attainment in Ireland [24]. Categorical variables were presented as counts and percentages, while continuous variables were presented as median [standard deviation (SD)] and range for non-parametric data, and as mean (SD) and range for normally distributed data. Statistical differences between CKD and non-CKD participants were assessed using the chi-square test for categorical variables, the *t*-test for normally distributed continuous variables and Wilcoxon-rank sum test for non-normally distributed continuous variables.

The dependent variable was CKD. Weighted multivariable logistic regression explored associations between traditional risk factors, emerging socioeconomic factors using the CKD-EPI 2012_(Scr-CysC)_ equation. Variables significant in the univariate analysis or perceived importance from literature were included. Model development was sequential: first exploring relationships of socioeconomic factors with CKD with demographic adjustment, then adding traditional risk factors and finally adding healthcare coverage (medical card/GP visit card). Adjusted ORs with 95% confidence intervals (CIs) were reported. Effect modification was tested with interaction terms, and model performance was assessed using the area under the curve of receiver operating characteristic (AUC-ROC) curve. A *P*-value of <.05 was considered statistically significant.

In sensitivity analysis, the modelling strategy was repeated using the CKD-EPI 2021_(Scr-CysC)_. These series of models followed a similar sequence to the process outlined above. Furthermore, all analysis were conducted with and without the inclusion of missing values. Statistical analysis were executed using the ‘survey’ package and eGFR calculated using ‘nephro’ [25] package with the R version 4.3.2 (2023-10-31 ucrt).

## RESULTS

### Cohort description

**Figure 1:**
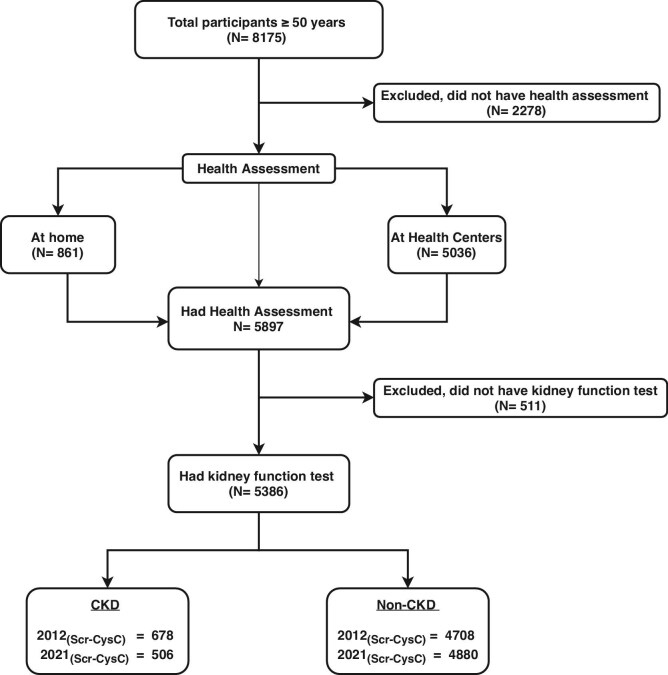
Strobe diagram of study participants from the TILDA cohort aged >50 years and older who completed a computer-assisted face-to-face interview in their home, a self-complete postal questionnaire and a centre- or home-based health assessment and have has serum creatinine and cystatin C measurements.

From 8175 participants aged 50 years and over, 5386 participants underwent assessment of kidney function (Fig. 1, STROBE diagram). The median age was 62 years, 51.1% were women, 37.2% had primary level of education, 35.4% were unemployed, 48.7% had a medical or GP visit card, and 12.3% were hospitalized in the previous 12 months (Table 1). Compared with participants without CKD, those with CKD were significantly older, women and included a significantly higher percentage of individuals with chronic disease and obesity. Moreover, those with CKD had significantly lower percentage completing secondary and tertiary education and higher percentage in unemployment or retirement. These individuals also had greater dependency on financial support through a medical or GP card and had more admissions to hospital within the previous 12 months.

**Table 1:** Baseline characteristics of TILDA participants by CKD status according to the CKD-EPI 2012_(Scr-CysC)_ equation.

			CKD-EPI 2012_(Scr-CysC)_				
	Total		No CKD		CKD		
	*N*	%	*n*	%	*n*	%	*P*-value
Overall	5386		4708	85.3	678	14.7	
Demographic characteristics							
Age, years (median, SD, R)	5386	62.0 (9.1), 50.0–80.0	4708	60.0 (8.2), 50.0–80.0	678	76.0 (7.4), 50.0–80.0	<.001
Age category							
50–64 years	3265	59.8	3156	67.7	109	14.2	<.001
65–74 years	1428	23.2	1188	22.4	240	27.9	
75+ years	693	17.0	364	9.9	329	57.9	
Sex							
Men	2505	48.9	2227	50.8	278	38.4	<.001
Women	2881	51.1	2481	49.2	400	61.6	
**Social and behavioural characteristics**							
Marital status							
Single (never married)	427	7.7	368	7.6	59	8.7	<.001
Married	3811	69.2	3434	72.6	377	49.4	
Separated/widow/divorced	1027	21.0	792	17.6	235	40.8	
Living with partner as married	121	2.1	114	2.2	7	1.1	
Education							
Primary	1376	37.2	1091	33.4	285	59.6	<.001
Secondary	2221	43.8	1975	46.1	246	30.7	
Tertiary	1789	19.0	1642	20.5	147	9.7	
Current employment status							
Employed	2079	37.1	2019	42.1	60	8.0	<.001
Unemployed	1271	35.4	1087	25.3	184	30.5	
Retired	1964	26.1	1542	31.2	422	59.8	
Unknown	72	1.4	60	1.3	12	1.7	
Smoker							
Current	841	16.8	751	17.4	90	13.2	.036
Never	2433	44.0	2136	43.8	297	44.8	
Past	2112	39.2	1821	38.8	291	42.0	
Health service utilization							
Medical or GP visit card	2336	48.7	1804	42.6	532	84.4	<.001
Hospital admission (in past 12 months)	651	12.3	515	10.9	136	20.4	<.001
Comorbid conditions							
Hypertension	3392	65.4	2801	61.5	591	88.1	<.001
Bone diseases	1825	34.1	1503	31.3	322	49.7	<.001
CVD	564	11.6	388	8.9	176	26.6	<.001
Urinary incontinence	713	13.2	581	11.8	132	21.3	<.001
Diabetes	442	8.9	332	7.5	110	16.8	<.001
COPD	662	12.7	557	12.1	105	16.2	.005
Cancer	330	6.3	260	5.5	70	10.9	<.001
Mental health problems	496	8.9	446	9.3	50	6.5	.016
Anthropometric measurement							
Hip circumference, cm (mean, SD, R)	5367	105.2 (9.7), 66.4–173.7	4694	104.6 (9.2), 66.5–173.7	673	108.6 (11.4), 84.0–169.0	<.001
Waist circumference, cm (mean, SD, R)	5365	96.1 (13.7), 58.5–163.0	4693	95.4 (13.6), 58.5–163.0	672	100.2 (14.0), 64.7–151.5	<.001
BMI, kg/m² (mean, SD, R)	5372	28.2 (4.6), 18.0–45.0	4699	28.1 (4.5), 18.0–45.0	673	29.7 (4.9), 18.0–45.0	<.001
BMI category							
Normal	1221	21.5	1109	22.4	112	16.7	<.001
Overweight	2334	43.4	2084	44.5	250	37.1	
Obesity	1817	35.1	1506	33.2	311	46.7	
Biomarkers							
Mean systolic BP (mean, SD, R)	5356	136.5 (19.9), 83.0–220.0	4689	135.6 (19.3), 83.5–220.0	667	141.8 (22.2), 89.5–218.0	<.001
Mean diastolic BP (mean, SD, R)	5356	82.5 (11.3), 51.5–132.0	4689	82.8 (11.2), 51.5–132.0	667	80.6 (12.1), 55.5–118.5	<.001
Blood cholesterol (mean, SD, R)	5374	5.1 (1.1), 1.8–12.2	4700	5.2 (1.1), 1.8–12.2	674	4.6 (1.1), 2.2–8.2	<.001
Blood HDL (mean, SD, R)	5374	1.5 (0.4), 0.5–3.8	4700	1.5 (0.4), 0.6–3.8)	674	1.4 (0.4), 0.6–2.9	<.001
Blood LDL (mean, SD, R)	5374	2.9 (0.9), 0.2–8.5	4700	2.9 (0.9), 0.1–8.5	674	2.5 (0.9), 0.7–6.1	<.001
Blood triglycerides (median, SD, R)	5374	1.5(1.1), 0.3–15.8	4700	1.5 (1.1), 0.3–15.8	674	1.6 (0.9), 0.4–6.6	<.001
HbA1c (mean, SD, R)	5330	33.0 (5.7), 17.0–94.0	4658	33.3 (5.5), 17.0–94.0	672	35.3 (6.5), 23.0–75.0	<.001

All the presented results are weighted ^a^non-normal distribution median reported, chi square test applied to categorical variables and *t*-test to numeric variables if follow normal distribution otherwise Wilcoxon rank sum test.

*P* < .05 indicates statistically significant.

R, range; HDL, high-density lipoprotein; LDL, low-density lipoprotein.

### Prevalence of CKD in population

**Figure 2:**
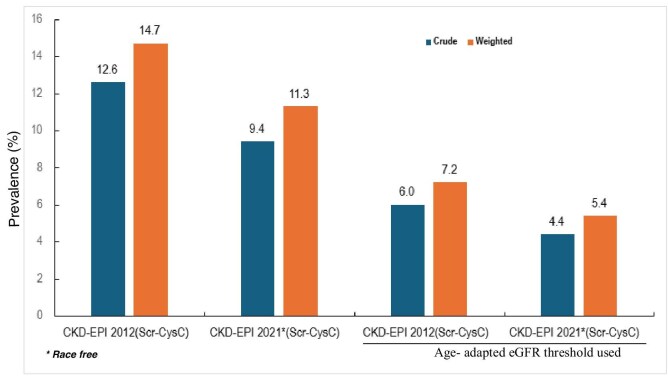
Crude and weighted prevalence of CKD among participants age >50 years provided by CKD-EPI 2012_(Scr-CysC)_, CKD-EPI 2021_(Scr-CysC)_ (race-free) and age-adapted equations The weighted values account for sample design complexities to provide a more accurate representation of the community-dwelling population aged 50 years and over.

**Figure 3:**
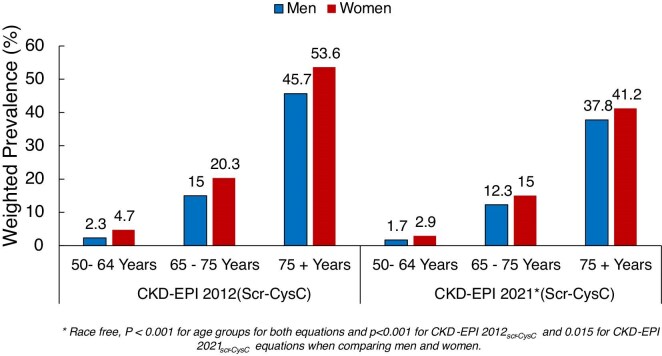
Weighted prevalence of CKD by age and sex using CKD-EPI 2012_(Scr-CysC)_ and CKD-EPI 2021_(Scr-CysC)_ equations. The asterisk indicates that the CKD-EPI 2021_(Scr-CysC)_ equation is race-free and *P*-value between age groups is <.001 for both equations, and between men and women is <.001 for CKD-EPI 2012_(Scr-CysC)_ and .015 for CKD-EPI 2021_(Scr-CysC)_ equations.

The weighted prevalence of CKD was 14.7% (95% CI 13.6–15.9) according to CKD-EPI 2012_(Scr-CysC)_ and 11.3% (95% CI 10.2–12.3) according to the CKD-EPI 2021_(Scr-CysC__)_, respectively, and these estimates were higher than the respective crude prevalence for each equation (Fig. 2). The prevalence of CKD increased with advancing age, and the prevalence in women exceeded that in men (Fig. 3). Within each age and sex category, the prevalence of CKD was significantly lower in CKD-EPI 2021_(Scr-CysC)_ equation compared with the CKD EPI 2012_(Scr-CysC)_ equation. The magnitude of these differences was greatest for older individuals and for women. Using the age-adapted eGFR thresholds to define CKD, the weighted CKD prevalence was lowered to 7.2% (95% CI 5.4–6.7) for the CKD-EPI 2012_(Scr-CysC)_, and to 5.4% (95% CI 4.6–6.1) for the CKD-EPI 2021_(Scr-CysC)_.

### Prevalence of CKD within demographic and socio-behavioural subgroups

Based on the CKD-EPI 2012_(Scr-CysC)_, the prevalence of CKD was higher in women than men (17.8% vs 11.5%), and higher in separated, widowed or divorced individuals compared with other marital categories (28.6% vs others) (Table 2). Participants with primary level education had significantly higher prevalence of CKD compared with others with education higher than primary. Similarly, CKD was far more common among those with a medical or GP visit card than without (25.5% vs 4.5%), and those hospitalized within the previous year (24.4% vs 13.4%).

**Table 2:** CKD prevalence by demographic and socio-behavioural characteristics of participants, based on CKD-EPI 2012_(Scr-CysC)_ and CKD-EPI 2021_(Scr-CysC)_.

		CKD-EPI 2012_(Scr-CysC)_		CKD-EPI 2021_(Scr-CysC)_^a^	
	Total (*N*)	Cases (*n*)	CKD prevalence, % (95% CI)	Cases (*n*)	CKD prevalence, % (95% CI)
Overall	5386	678	14.7 (13.6–15.9)	506	11.3 (10.2–12.3)
Demographic characteristics					
Age category*					
50–64 years	3265	109	3.5 (2.8–4.1)	71	2.3 (1.8–2.8)
65–74 years	1428	240	17.6 (15.4–19.9)	180	13.6 (11.7–15.5)
75+ years	693	329	50.3 (46.3–54.3)	255	39.8 (35.8–43.8)
Sex*					
Men	2505	278	11.5 (10.3–12.8)	220	9.4 (8.2–10.6)
Women	2881	400	17.8 (16.0–19.5)	286	13.1 (11.6–14.6)
Social and behavioural characteristics					
Marital status*					
Single (never married)	427	59	16.5 (12.4–20.6)	50	14.3 (10.8–18.7)
Married	3811	377	10.5 (9.3–11.6)	270	7.7 (6.8–8.8)
Separated/widow/divorced	1027	235	28.6 (25.3–31.8)	182	22.6 (19.7–25.9)
Living with partner as married	121	7	8.2 (1.3–15.0)	4	3.9 (1.4–10.5)
Education*					
Primary	1376	285	23.6 (21.2–26.0)	224	18.6 (16.3–20.9)
Secondary	2221	246	10.3 (8.9–11.7)	187	7.8 (6.7–9.0)
Tertiary	1789	147	7.5 (6.2–8.8)	95	4.8 (3.8–5.8)
Current employment status*					
Employed	2079	60	3.2 (2.3–4.0)	38	2.1 (1.4–2.7)
Unemployed	1271	184	17.2 (14.7–19.7)	127	12.5 (10.2–14.8)
Retired	1964	422	24.8 (22.6–27.1)	331	19.8 (17.7–21.9)
Unknown	72	12	18.5 (8.2–28.8)	10	16.4 (6.2–26.6)
Smoker**					
Current	841	90	11.6 (9.1–13.9)	64	8.3 (6.2–10.3)
Never	2433	297	15.0 (13.1–16.9)	215	11.6 (9.9–13.2)
Past	2112	291	15.8 (13.9–17.5)	227	12.3 (10.7–13.8)
Health service utilization					
Medical/GP card*					
No	3050	146	4.5 (3.7–5.2)	92	3.0 (2.3–3.5)
Yes	2336	532	25.5 (23.5–27.5)	414	20.1 (18.2–21.9)
Hospital admission* (within the past 12 months)					
No	4735	542	13.4 (12.2–14.6)	398	10.1 (9.0–11.1)
Yes	651	136	24.4 (20.8–28.0)	108	19.9 (16.5–23.4)

^a^Race-free GFR equation.

*P*-value calculated using chi-square test for categorical variables.

*P-value <.001, **P-value <.05 for both equations.

See Supplementary data, Table S1 for weighted and unweighted prevalence estimates.

### Prevalence of CKD among participants with chronic medical conditions

The prevalence of CKD was significantly higher in participants with major chronic conditions compared with those without using the CKD-EPI 2012_(Scr-CysC)_, with the greatest burden observed in those with CVD (33.9%), diabetes (28.0%), cancer (25.5%), urinary incontinence (23.7%) and bone diseases (21.5%). The prevalence of CKD was equally common among participants with hypertension (19.8%), obesity (19.5%) and COPD (18.8%), all of which were above the national average of 14.7%. Use of the CKD-EPI 2021_(Scr-CysC)_ yielded similar trends but the prevalence estimates were significantly lower (Table 3).

**Table 3:** Prevalence of CKD by chronic medical conditions of participants, based on CKD-EPI 2012_(Scr-CysC)_ and CKD-EPI 2021_(Scr-CysC)._^a^

		CKD-EPI 2012_(Scr-CysC)_		CKD-EPI 2021_(Scr-CysC)_^b^	
Chronic medical conditions	Total	Cases (*n*)	CKD prevalence % (95% CI)	Cases (*n*)	CKD prevalence % (95% CI)
CVD*	564	176	33.9 (29.8–38.0)	144	28.6 (24.5–32.6)
Diabetes*	442	110	28.0 (23.1–32.8)	95	24.4 (19.8–28.9)
Cancer*	330	70	25.5 (19.6–31.3)	57	21.0 (15.3–26.6)
Urinary incontinence*	713	132	23.7 (19.6–27.7)	98	18.1 (14.3–21.9)
Bone diseases*	1825	322	21.5 (19.2–23.8)	229	15.7 (13.7–17.7)
Hypertension*	3392	591	19.8 (18.3–21.4)	450	15.5 (14.0–16.9)
Obesity*	1817	311	19.5 (17.3–21.6)	245	15.7 (13.7–17.6)
COPD**	662	105	18.8 (15.3–22.4)	81	14.3 (11.2–17.3)
Mental health problems**	496	50	10.7 (7.8–13.7)	35	7.7 (5.1–10.3)

^a^The prevalence estimates were weighted (weighted prevalence) according to age, sex, and educational attainment in Ireland adjusted for selection and non-response bias in the health assessment component of the survey.

^b^Race-free eGFR equation.

COPD included chronic bronchitis, emphysema and asthma.

Bone disease included arthritis-osteoarthritis or rheumatism and osteoporosis.

Mental health problem included any emotional, nervous or psychiatric problem such as depression or anxiety including serious memory impairment.

*P*-value for chi-square test for comparisons of categorical variables.

*P-value of <.001, **P-value <.05 for both equations.

See Supplementary data, Table S2 for weighted and unweighted prevalence for both equations.

### Medication utilization among participants with CKD

The majority (94.2%) of participants with CKD were receiving at least one medication (excluding supplements), and 48.8% were treated with polypharmacy, defined as the use of five or more than five different medications. Treatment with either an angiotensin-converting enzyme inhibitor or angiotensin-receptor blocker was more common in CKD participants (47.2% vs 21.5, *P* < .001) while use of non-steroidal anti-inflammatory drugs was significantly common in those with CKD than without CKD (9.3% vs 6.1%, *P* < .005) Table 4.

**Table 4:** Medication use among individuals with CKD and non-CKD, based on CKD-EPI 2012_(Scr-CysC)_ (*N* = 5368).

		No CKD (*N* = 4708)		CKD (*N* = 678)	
	Total	*n*	%	*n*	%
On any medicine incl. supplements*	3828	3198	68.4	630	95.1
On medicine excl. supplements*	3645	3022	65.1	623	94.2
1–2 medicines*	1715	1568	50.0	147	22.0
3–4 medicines	1034	849	28.6	185	29.3
≥5 medicines (polypharmacy)	896	605	21.4	291	48.8
On supplements only**	1021	864	17.4	157	22.9
Types of medicines					
On any statin*	1643	1325	28.4	318	47.1
On ACE or ARB*	1279	961	21.5	381	47.2
On antihypertensive drugs*	780	548	12.4	232	34.9
On any ACE inhibitors*	681	512	11.9	169	25.3
On ARB*	620	463	9.9	157	23.0
On any NSAID**	340	279	6.1	61	9.3
On any blood glucose lowering drug—excl. insulin*	273	200	4.6	73	10.6

All the presented results are weighted.

*P*-value calculated applying chi-square test for comparisons of CKD and non-CKD groups.

*P-value of <.001,

**P-value of <.05.

See Supplementary data, Tables S3 and S4 for more detail on medication use among the participants by CKD, based on both CKD-EPI 2012_(Scr-CysC)_ and CKD-EPI 2021_(Scr-CysC)_ equations.

ARB, angiotensin II antagonist; ACE, angiotensin-converting enzymes; NSAID, non-steroidal anti-inflammatory drugs.

### Multivariable analysis of factors associated with prevalent CKD

The relationships of demographic, socio-behavioural, economic factors and clinical conditions with CKD are presented in sequential multivariable models (Table 5). In the fully adjusted model (Model 3), each 1-year increase in age over 50 years, was associated with greater odds of having CKD [OR 1.15 (95% CI 1.13–1.17)]. Women [OR 1.54 (1.25–1.90)], unemployed [OR 1.86 (95% CI 1.28–2.70)] and participants with a medical or GP card had greater likelihood of having CKD.

**Table 5:** Univariate and multivariate logistic regression models of factors associated with prevalent CKD based on CKD-EPI 2012_(Scr-CysC)._

			Multivariate logistic regression					
	Univariate logistic regression		Model 1 (*N* = 5384)		Model 2 (*N* = 5306)		Model 3 (*N* = 5306)	
Characteristics	OR (95% CI)	*P*-value	AOR (95% CI)	*P*-value	AOR (95% CI)	*P*-value	AOR (95% CI)	*P*-value
Age	1.18 (1.16–1.19)	<.001	1.17 (1.15–1.18)	<.001	1.16 (1.14–1.18)	<.001	1.15 (1.13–1.17)	<.001
Sex								
Men	1.00		1.00		1.00		1.00	
Women	1.65 (1.40–1.95)	<.001	1.31 (1.07–1.60)	.008	1.51 (1.22–1.87)	<.001	1.51 (1.22–1.87)	<.001
Education								
Tertiary	1.00		1.00		1.00		1.00	
Primary	3.79 (3.01–4.79)	<.001	1.51 (1.17–1.96)	.001	1.28 (0.98–1.67)	.065	1.19 (0.91–1.56)	.196
Secondary	1.42 (1.12–1.79)	.003	1.28 ( 0.99–1.65)	.062	1.16 (0.89–1.51)	.253	1.10 (0.85–1.44)	.453
Employment								
Employed	1.00		1.00		1.00		1.00	
Retired	10.14 (7.43–13.83)	<.001	1.55 (1.09–2.21)	<.015	1.45 (1.01–2.10)	.046	1.37 (0.95–1.97)	.096
Unemployed	6.41 (4.62–8.90)	<.001	2.17 (1.52–3.11)	<.001	2.01 (1.39–2.90)	<.001	1.86 (1.28–2.70)	.001
CVD								
No	1.00				1.00		1.00	
Yes	3.69 (2.97–4.59)	<.001			1.50 (1.16–1.93)	.001	1.42 (1.10–1.84)	.008
Diabetes								
No	1.00				1.00		1.00	
Yes	2.49 (1.92–3.24)	<.001			1.51 (1.10–2.06)	.009	1.45 (1.06–2.00	.019
Hypertension								
No	1.00				1		1	
Yes	4.68 (3.62–6.05)	<.001			1.77 (1.35–2.34)	<.001	1.78 (1.35–2.35)	<.001
COPD								
No	1.00				1.00		1.00	
Yes	1.41 (1.11–1.80)	.005			1.25 (0.96–1.63)	.097	1.19 (0.91–1.56)	.203
Cancer								
No	1.00				1.00		1.00	
Yes	2.10 (1.52–2.89)	<.001			1.58 (1.11–2.25)	.011	1.53 (1.08–2.18)	.017
Bone disease								
No	1.00				1.00		1.00	
Yes	2.16 (1.79–2.61)	<.001			1.10 (0.88–1.37)	.398	1.09 (0.87–1.36)	.466
BMI								
Normal	1.00				1.00		1.00	
Overweight	1.16 (0.89–1.50)	.276			1.36 (1.01–1.85)	.045	1.37 (1.01–1.85)	.041
Obesity	1.95 (1.50–2.54)	<.001			2.34 (1.72–3.20)	<.001	2.32 (1.70–3.15)	<.001
Hospital admission (within the past 12 months)								
No	1.00						1.00	
Yes	2.09 (1.69–2.60)	<.001					1.50 (1.13–1.9)	.004
Medical or GP card								
No	1.00						1.00	
Yes	7.31 (5.96–8.96)	<.001					1.31 (1.03–1.66)	.024
AUC-ROC			0.84		0.85		0.86	

Note: all the presented results are weighted.

Model 1: demographic and socioeconomic factors.

Model 2: model 1 plus traditional risk factors.

Model 3: model 2 plus healthcare coverage, and recent hospitalizations.

COPD included chronic bronchitis, emphysema, asthma.

BMI categories: normal (18.5–24.9 kg/m^2^), overweight (BMI 25 –29.9 kg/m2), obesity (BMI ≥30 kg/m^2^).

AOR, adjusted OR.

**Figure 4:**
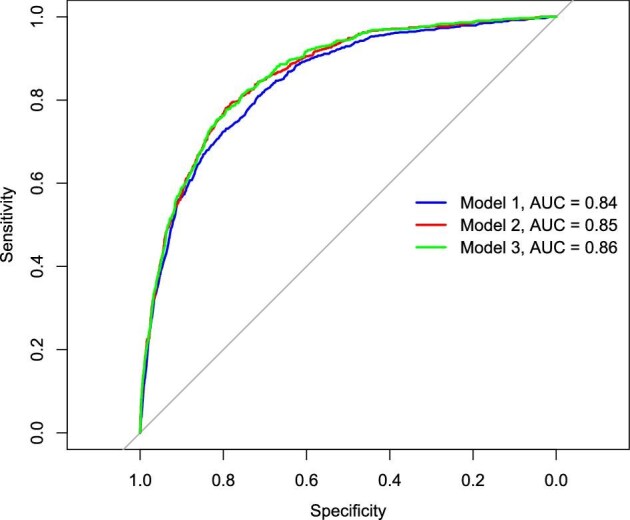
The AUC-ROC comparing logistic regression models’ performance to determine factors associated with CKD. The curves illustrate the sensitivity versus specificity for each model, with the AUC values indicating the model performance. Model 1 (blue) achieved an AUC of 0.84, Model 2 (red) achieved an AUC of 0.85 and Model 3 (green) achieved an AUC of 0.86. The diagonal line represents the line of no-discrimination, where the model performs no better than random chance.

The model also identified several chronic diseases: hypertension [OR 1.78 (95% CI 1.35–2.35)], diabetes [OR 1.45 (95% CI 1.06–1.99)], cancer [OR 1.53, CI 1.08–2.18)] and CVD [OR 1.43 (95% CI 1.10–1.85)] as significant correlates. BMI exhibited a graded relationship, with increasing likelihood of CKD for overweight and obese individuals [OR 1.37 (95% CI 1.01–1.85) and 2.33 (95% CI 1.71–3.17), respectively]. Additionally, the likelihood of having CKD was higher for participants who had been hospitalized in the previous year [OR 1.5 (95% CI 1.1–2.0)]. Model performance, using the AUC ranged from 0.84 to 0.86, as shown in Fig. 4.

### Sensitivity analysis and interactions

In sensitivity analysis, we replicated the modelling strategy using CKD-EPI 2021_(Scr-CsyC)_ equation to estimate eGFR. The results generated from these models were identical to original analysis (Supplementary data, Table S5). Furthermore, the inclusion or exclusion of variables with missing values did not significantly alter model performance. We also tested for interactions between age and sex, and comorbid conditions, but these did not reveal any strong combined effects on CKD.

## DISCUSSION

In this national study of adults aged 50 years and older, we provide population-based estimates of CKD prevalence and its determinants in the Irish population. We report a weighted prevalence of 14.7% for CKD based on the CKD-EPI 2012_(Scr-CysC)_ among community dwelling individuals that was substantially higher in older women and men. The high prevalence of CKD was observed not only in traditional high-risk groups such as those with CVD, diabetes and hypertension but also in several emerging risk groups including individuals with cancer, overweight and obesity, bone disease and COPD. Furthermore, multivariable analysis identified educational achievement, employment status and access to healthcare as independent correlates of CKD, and highlights the increasing importance of the socioeconomic environment in disease burden. Targeting these high-risk phenotypes in our health system through national surveillance programmes are likely to yield substantial benefits through effective disease management and proactive population health planning.

Our study generated precise estimates of CKD burden in Ireland and pinpointed the most affected subgroups from the TILDA cohort. We found that individuals with preexisting CVD disease, diabetes and hypertension were in the highest risk groups with CKD prevalence in excess of 20%, findings that are consistent to international studies [26, 27]. We also reveal that individuals with cancer, bone diseases and obesity had a CKD burden that was similar to or greater than those with hypertension. Studies of cancer cohorts have reported prevalence estimates of CKD between 12% and 25%, which vary according to the type of cancer [28–30]. Similarly, studies of arthritis populations have yielded estimates that vary between 7% and 24% [31–33]. Obesity has been strongly linked to incident CKD [34–36], although precise estimations of CKD burden in obese populations are limited. In TILDA, almost 20% of obese individuals had CKD and our analysis revealed a gradient effect with increasing likelihood of CKD observed for overweight and obese individuals [37].

Our study lends further support to the observations from Jonsson *et al*. who found direct associations between obesity and cancer with incident CKD suggesting that these conditions may exert a causative role [38]. Taken together, these would suggest that a multi-faceted approach that targets traditional and emerging risk factors is required to prevent CKD and downstream complications.

Our analysis provides compelling evidence of variation in CKD burden across demographic and socioeconomic domains, with a 40-fold increase in prevalence from ages 50–64 to 75+ years for both men and women, highlighting implications for national healthcare planning. In line with previous studies [39–41], our analysis demonstrates higher CKD prevalence in women than men, with this difference persisting into the older age. The reasons for these differences remain unclear but may in part reflect differences in the pathophysiology of CKD between men and women, treatment provision, health behaviours and eGFR estimation [42, 43]. Women in TILDA were >50% more likely to have CKD taking into consideration of known risk factors including diabetes, hypertension, obesity and several socioeconomic factors.

The contribution of social inequality to CKD prevalence was apparent from our analysis with unemployed individuals having more than a 5-fold higher prevalence than those employed, stressing the need to address these disparities in preventative strategies. This relationship persisted with adjustment for comorbid conditions, medical card use and education. Similarly, education was identified as an important correlate of CKD. Individuals with primary level education experienced a far higher prevalence than those with third-level education (23.6% vs 7.5%). Those on free or subsidized healthcare also had a higher burden of CKD supporting the evidence that lower socioeconomic status is directly connected to CKD through an excess of adverse risk factors [44–46]. Although the possibility of reverse causality may exist—with CKD contributing to unemployment and worsening socioeconomic conditions—our observations point to social inequality as a strong correlate of CKD, likely driven by disparities in healthcare access and lifestyle-related factors. The co-existence of a potential bidirectional relationship highlights the importance of public health strategies that consider socioeconomic status as a potential cause and consequence of CKD.

At the population level, we observed a 3% difference in prevalence between the CKD-EPI 2012_(Scr-CysC)_ and the race-free CKD-EPI 2021_(Scr-CysC)_ (14.7% vs 11.3%). The adoption of the CKD-EPI 2021_(Scr-CysC)_ would therefore lower the CKD population prevalence by nearly 40 000 individuals. Similarly, adoption of the newly proposed age-adapted CKD definition for population health planning would further reduce the absolute prevalence by an even greater percentage ranging from 5.9% (CKD-EPI 2021_(Scr-CysC)_ 11.3% minus 5.4%) to 7.5% (CKD-EPI 2021_(Scr-CysC)_ 14.7% minus 7.2%) [23]. If Ireland adopted the CKD-EPI 2021_(Scr-CysC)_ or the age-adapted CKD definition rather than the CKD-EPI 2012_(Scr-CysC)_ as the gold standard, then strategic planning of renal services, resource allocation and prevention strategies would be based on estimates of CKD that are far lower than currently estimated. Equally important at the individual level, the higher values of eGFR derived from CKD-EPI 2021_(Scr-CysC)_ compared with CKD-EPI 2012_(Scr-CysC)_ would lead to a net reclassification of KDIGO stage and corresponding level of risk [47], with important downstream consequences on clinical decision-making that has direct implications for referral rates, extent of investigation and treatment strategies. The generation of country-specific equations that take into consideration the natural age-related decline in GFR and the expected differences between men and women excluding race may help to resolve these discrepancies [47].

This study has limitations, including its cross-sectional design, reliance on self-reported data, single-time-point measurements of creatinine and cystatin C, all of which may lead to imprecision [47]. Furthermore, the absence of urine albumin-to-creatinine ratio restricts a more comprehensive assessment of kidney function and CKD staging. We recommend inclusion of urine testing for albuminuria in future iterations of TILDA to enable more accurate assessment of CKD, as well as follow-up measurements of serum creatinine in a subsample that would extend beyond 3 months to better align with the KIDGO criteria for CKD diagnosis. Despite this, it has several key strengths: TILDA is a nationally representative study of adults aged 50+ years, providing comprehensive demographic, socioeconomic, clinical and treatment data [16, 17]. Our analysis generated population-level estimates of CKD prevalence by applying sample weights to account for the complex study design and compared contemporary equations for estimating eGFR: the CKD-EPI 2021_(Scr-CysC)_ compared with the CKD-EPI 2012_(Scr-CysC)_. The modelling strategy explored the relative contribution of several factors to CKD burden.

In conclusion, compared with the national average, the burden of CKD is far greater in older individuals with major chronic diseases and the socioeconomically disadvantaged. Beyond the traditional high-risk groups, CKD is highly prevalent in disease groups with cancer, obesity, COPD and bone diseases. The contribution of the socioeconomic environment to CKD burden is substantial and is dependent on level of educational attainment, unemployment and access to healthcare. The identification and targeting of these groups through national surveillance programmes is likely to yield substantial benefits from more effective disease management and proactive population health planning.

## Supplementary Material

sfaf065_Supplemental_File

## Data Availability

The data used in this study was publicly available anonymised dataset from TILDA that can be retrieved through the Irish Social Science Data Archive at www.ucd.ie/issda [19].
