# Supplementary material for: Prevalence and determinants of chronic kidney disease among community-dwelling adults, 50 years and older in Ireland
Source: Clin Kidney J. 2025 Mar 11;18(3):sfaf065. doi: 10.1093/ckj/sfaf065 (PMC11932339; doi:10.1093/ckj/sfaf065)
Supplement: sfaf065_Supplemental_File [file sfaf065_supplemental_file.docx]

**Supplementary Table 1: CKD prevalence by demographic and socio-behavioural characteristics, based on CKD-EPI 2012_(Scr-CysC)_ and CKD-EPI 2021_(Scr-CysC)_**

|  | **Total** | | | **CKD, based on CKD-EPI 2012_(Scr-CysC)_** | | | | | | | | **CKD, based on CKD-EPI 2021_(Scr-CysC)_ ^b^** | | | | | | | |
| --- | --- | --- | --- | --- | --- | --- | --- | --- | --- | --- | --- | --- | --- | --- | --- | --- | --- | --- | --- |
|  | **N** | **UW**  **(%)** | **W**  **(%)** | **No (n)** | **Yes (n)** | **UW** | | | **W** | | | **No (n)** | **Yes (n)** | **UW** | | | **W** | | |
|  |  |  |  |  |  | **No**  **(%)** | **Yes**  **(%)** | **p-value** | **No**  **(%)** | **Yes (%)** | **p-value** |  |  | **No (%)** | **Yes (%)** | **p-value** | **No (%)** | **Yes (%)** | **p-value** |
| **Overall** | **5386** |  |  | **4708** | **678** | **87.4** | **12.6** |  | **85.3** | **14.7** |  | **4880** | **506** | **90.6** | **9.4** |  | **88.7** | **11.3** |  |
| **Demographic characteristics** | | |  |  |  |  |  |  |  |  |  |  |  |  |  |  |  |  |  |
| **Age in years**  (mean/md, SD, R) ^a^ | 5386 | 62.0 (8.7), 50.0 -80.0 | 62.0 (9.1),  50.0-80.0 | 4708 | 678 | 60.0 (8.0), 50.0 - 80.0 | 74.0 (7.5), 50.0 -80.0 | <0.001 | 60.0 (8.2), 50.0 – 80.0 | 76.0 (7.4), 50.0 – 80.0 | <0.001 | 4880 | 506 | 60.0 (8.2), 50.0 - 80.0 | 75.0 (7.3), 50.0 – 80.0 | <0.001 | 61.0 (8.5),  50.0-80.0 | 77.0 (7.2),  50.0-80.0 | <0.001 |
| **Age category** |  |  |  |  |  |  |  |  |  |  |  |  |  |  |  |  |  |  |  |
| 50-64 years | 3265 | 60.6 | 59.8 | 3156 | 109 | 96.7 | 3.3 | <0.001 | 96.5 | 3.5 | <0.001 | 3194 | 71 | 67.1 | 2.2 | <0.001 | 97.7 | 2.3 | <0.001 |
| 65-74 years | 1428 | 26.5 | 23.2 | 1188 | 240 | 83.2 | 16.8 |  | 82.4 | 17.6 |  | 1248 | 180 | 87.4 | 12.6 |  | 86.4 | 13.6 |  |
| 75+ years | 693 | 12.9 | 17.0 | 364 | 329 | 52.3 | 47.5 |  | 49.7 | 50.3 |  | 438 | 255 | 97.8 | 36.8 |  | 60.2 | 39.8 |  |
| **Sex** |  |  |  |  |  |  |  |  |  |  |  |  |  |  |  |  |  |  |  |
| Men | 2505 | 46.5 | 48.9 | 2227 | 278 | 88.9 | 11.1 | 0.002 | 88.5 | 11.5 | <0.001 | 2285 | 220 | 91.2 | 8.8 | 0.164 | 90.6 | 9.4 | <0.001 |
| Women | 2881 | 53.5 | 51.1 | 2481 | 400 | 86.1 | 13.9 |  | 82.2 | 17.8 |  | 2595 | 286 | 90.1 | 9.9 |  | 86.9 | 13.1 |  |
| **Social and behavioural characteristics** | | | | |  |  |  |  |  |  |  |  |  |  |  |  |  |  |  |
| **Marital status** |  |  |  |  |  |  |  |  |  |  |  |  |  |  |  |  |  |  |  |
| Single (Never married) | 427 | 7.9 | 7.7 | 368 | 59 | 86.2 | 13.8 | <0.001 | 83.5 | 16.5 | <0.001 | 377 | 50 | 88.3 | 11.7 | <0.001 | 85.7 | 14.3 | <0.001 |
| Married | 3811 | 70.8 | 69.2 | 3434 | 377 | 90.1 | 9.9 |  | 89.5 | 10.5 |  | 3541 | 270 | 92.9 | 7.1 |  | 92.3 | 7.7 |  |
| Separated/widow/divorced | 1027 | 19.1 | 21.0 | 792 | 235 | 77.1 | 22.9 |  | 71.4 | 28.6 |  | 845 | 182 | 82.3 | 17.7 |  | 77.4 | 22.6 |  |
| Living with partner as married | 121 | 2.2 | 2.1 | 114 | 7 | 94.2 | 5.8 |  | 91.8 | 8.2 |  | 117 | 4 | 96.7 | 3.3 |  | 96.1 | 3.9 |  |
| **Education** |  |  |  |  |  |  |  |  |  |  |  |  |  |  |  |  |  |  |  |
| Primary | 1376 | 25.5 | 37.2 | 1091 | 285 | 79.3 | 20.7 | <0.001 | 76.4 | 23.6 | <0.001 | 1152 | 224 | 83.7 | 16.3 | <0.001 | 81.4 | 18.6 | <0.001 |
| Secondary | 2221 | 41.3 | 43.8 | 1975 | 246 | 88.9 | 11.1 |  | 89.7 | 10.3 |  | 2034 | 187 | 91.6 | 8.4 |  | 92.1 | 7.9 |  |
| Tertiary | 1789 | 33.2 | 19.0 | 1642 | 147 | 91.8 | 8.2 |  | 92.5 | 7.5 |  | 1694 | 95 | 94.7 | 5.3 |  | 95.2 | 4.8 |  |
| **Current employment status** | | |  |  |  |  |  |  |  |  |  |  |  |  |  |  |  |  |  |
| Employed | 2079 | 38.6 | 37.1 | 2019 | 60 | 97.1 | 2.9 | <0.001 | 96.8 | 3.2 | <0.001 | 2041 | 38 | 98.2 | 1.8 | <0.001 | 97.9 | 2.1 | <0.001 |
| Unemployed | 1271 | 23.6 | 35.4 | 1087 | 184 | 85.5 | 14.5 |  | 82.8 | 17.2 |  | 1144 | 127 | 90.0 | 10.0 |  | 87.5 | 12.5 |  |
| Retired | 1964 | 36.5 | 26.1 | 1542 | 422 | 78.5 | 21.5 |  | 75.2 | 24.8 |  | 1633 | 331 | 83.1 | 16.9 |  | 80.2 | 19.8 |  |
| Unknown | 72 | 1.3 | 1.4 | 60 | 12 | 83.3 | 16.7 |  | 81.5 | 18.5 |  | 62 | 10 | 86.1 | 13.9 |  | 83.6 | 16.4 |  |
| **Smoker** |  |  |  |  |  |  |  |  |  |  |  |  |  |  |  |  |  |  |  |
| Current | 841 | 15.6 | 16.8 | 751 | 90 | 89.3 | 10.7 | 0.056 | 88.4 | 11.6 | 0.036 | 777 | 64 | 92.4 | 7.6 | 0.013 | 91.7 | 8.3 | **0.022** |
| Never | 2433 | 45.2 | 44.0 | 2136 | 297 | 87.8 | 12.2 |  | 85.0 | 15.0 |  | 2218 | 215 | 91.2 | 8.8 |  | 88.4 | 11.6 |  |
| Past | 2112 | 39.2 | 39.2 | 1821 | 291 | 86.2 | 13.8 |  | 84.2 | 15.8 |  | 1885 | 227 | 89.3 | 10.7 |  | 87.7 | 12.3 |  |
| **Health service utilisation** | |  |  |  |  |  |  |  |  |  |  |  |  |  |  |  |  |  |  |
| **Medical/GP card** |  |  |  |  |  |  |  |  |  |  |  |  |  |  |  |  |  |  |  |
| **No** | 3050 | 56.6 | 51.3 | 2904 | 146 | 95.2 | 4.8 | <0.001 | 95.5 | 4.5 | <0.001 | 2958 | 92 | 97.0 | 3.0 | <0.001 | 97.1 | 3.0 | <0.001 |
| **Yes** | 2336 | 43.4 | 48.7 | 1804 | 532 | 77.2 | 22.8 |  | 74.5 | 25.5 |  | 1922 | 414 | 82.3 | 17.7 |  | 79.9 | 20.1 |  |
| **Hospital admission**  (in past 12 months) |  |  |  |  |  |  |  |  |  |  |  |  |  |  |  |  |  |  |  |
| **No** | 4735 | 87.9 | 87.7 | 4193 | 542 | 88.6 | 11.5 | <0.001 | 86.6 | 13.4 | <0.001 | 4337 | 398 | 91.6 | 8.4 | <0.001 | 89.9 | 10.1 | <0.001 |
| **Yes** | 651 | 12.1 | 12.3 | 515 | 136 | 79.1 | 20.9 |  | 75.6 | 24.4 |  | 543 | 108 | 83.4 | 16.6 |  | 80.1 | 19.9 |  |
| *^a^ Data non-normal distribution, P- value calculated applying chi-square test to categorical variables and t-test to numeric variables if follow normal distribution otherwise Wilcoxon rank sum test. Comparison made among each category (example for sex, compared between men and women, age- among different age groups- 50-64 years, 65- 74 years, and >75 years and so on for other variables). P < 0.05 indicates statistically, significant. UW- unweighted, W- weighted,* **^b^***race free* | | | | | | | | | | | | | | | | | | | |

**Supplementary Table 2: Prevalence of CKD by chronic medical conditions of participants based on CKD-EPI 2012_(Scr-CysC)_ and CKD-EPI 2021_(Scr-CysC)_**

|  | **Total (N=5386)** | | | **CKD, based on CKD-EPI 2012_(Scr-CysC)_** | | | | | | | | **CKD, based on CKD-EPI 2021_(Scr-CysC)_ ^b^** | | | | | | | |
| --- | --- | --- | --- | --- | --- | --- | --- | --- | --- | --- | --- | --- | --- | --- | --- | --- | --- | --- | --- |
|  |  |  |  | **No**  **(n)** | **Yes**  **(n)** | **UW** | | | **W** | | |  |  | **UW** | | | **W** | | |
|  | **n** | **UW**  **%** | **W**  **(%)** |  |  | **No**  **(%)** | **Yes**  **(%)** | **P value** | **No**  **(%)** | **Yes**  **(%)** | **P value** | **No**  **(n)** | **Yes**  **(n)** | **No**  **(%)** | **Yes**  **(%)** | **P value** | **No**  **(%)** | **Yes**  **(%)** | **P value** |
| Cardiovascular disease | 564 | 10.5 | 11.6 | 388 | 176 | 68.8 | 31.2 | <0.001 | 66.1 | 33.9 | <0.001 | 420 | 144 | 74.5 | 25.5 | <0.001 | 71.4 | 28.6 | <0.001 |
| Diabetes | 442 | 8.3 | 8.9 | 332 | 110 | 75.1 | 24.9 | <0.001 | 72.0 | 28.0 | <0.001 | 347 | 95 | 78.5 | 21.5 | <0.001 | 75.6 | 24.4 | <0.001 |
| Cancer | 330 | 6.1 | 6.3 | 260 | 70 | 78.8 | 21.2 | <0.001 | 74.5 | 25.5 | <0.001 | 273 | 57 | 5.6 | 17.3 | <0.001 | 79.0 | 21.0 | <0.001 |
| Urinary incontinence | 713 | 13.2 | 13.2 | 581 | 132 | 81.5 | 18.5 | <0.001 | 76.3 | 23.7 | <0.001 | 615 | 98 | 86.3 | 13.7 | <0.001 | 81.9 | 18.1 | <0.001 |
| Bone diseases | 1825 | 33.9 | 34.1 | 1503 | 322 | 82.4 | 17.6 | <0.001 | 78.5 | 21.5 | <0.001 | 1596 | 229 | 87.4 | 12.6 | <0.001 | 84.3 | 15.7 | <0.001 |
| Hypertension | 3392 | 63.1 | 65.4 | 2801 | 591 | 82.6 | 17.4 | <0.001 | 80.2 | 19.8 | <0.001 | 2942 | 450 | 86.7 | 13.3 | <0.001 | 84.5 | 15.5 | <0.001 |
| Obesity | 1817 | 33.8 | 35.1 | 311 | 1506 | 82.9 | 17.1 | <0.001 | 80.5 | 19.5 | <0.001 | 1572 | 245 | 86.5 | 13.5 | <0.001 | 84.3 | 15.7 | <0.001 |
| COPD | 662 | 12.3 | 12.7 | 557 | 105 | 84.1 | 15.9 | 0.008 | 81.2 | 18.8 | 0.005 | 581 | 81 | 87.8 | 12.2 | 0.009 | 85.7 | 14.3 | 0.021 |
| Mental health problems | 496 | 9.2 | 8.9 | 446 | 50 | 89.9 | 10.1 | 0.089 | 89.3 | 10.7 | 0.016 | 461 | 35 | 92.9 | 7.1 | 0.073 | 92.3 | 7.7 | 0.020 |

*UW- unweighted, W- weighted, brace free, Chronic Obstructive Pulmonary Disease, included chronic bronchitis, emphysema, asthma; bone disease included arthritis-osteoarthritis or rheumatism and osteoporosis; mental health problem included any emotional, nervous, or psychiatric problem such as depression or anxiety including serious memory impairment. P- value calculated applying chi-square test comparing participants with and without comorbidities (example- with CVD vs. without CVD). P < 0.05 indicates statistically significant.*

**Supplementary Table 3: Medication among the participants by CKD, based on CKD-EPI 2012** _(Scr-CysC)_ **and CKD-EPI 2021** _(Scr-CysC)_

|  | **Total** | | | **CKD, based on CKD-EPI 2012**_(Scr-CysC)_ | | | | | | | | **CKD, based on CKD-EPI 2021** _(Scr-CysC)_ | | | | | | | |
| --- | --- | --- | --- | --- | --- | --- | --- | --- | --- | --- | --- | --- | --- | --- | --- | --- | --- | --- | --- |
|  | **N** | **UW**  **(%)** | **W**  **(%)** | **No (n)** | **Yes (n)** | **UW** | | | **W** | | | **No (n)** | **Yes (n)** | **UW** | | | **W** | | |
|  |  |  |  |  |  | **No**  **(%)** | **Yes**  **(%)** | **P -value** | **No (%)** | **Yes (%)** | **p-value** |  |  | **No (%)** | **Yes (%)** | **p-value** | **No (%)** | **Yes (%)** | **p-value** |
| **Overall** | 5386 |  |  | 4708 | 678 |  |  |  |  |  |  | **4880** | **506** |  |  |  |  |  |  |
| On any medicine incl. supplements | 3828 | 71.5 | 72.3 | 3198 | 630 | 68.2 | 94.5 | <0.001 | 68.4 | 95.1 | <0.001 | 3356 | 472 | 69.1 | 95.3 | <0.001 | 69.4 | 95.8 | <0.001 |
| **On medicine excl. supplements** | 3645 | 68.1 | 69.3 | 3022 | 623 | 64.5 | 93.4 | <0.001 | 65.1 | 94.2 | <0.001 | 3176 | 469 | 65.4 | 94.7 | <0.001 | 66.1 | 95.2 | <0.001 |
| 1-2 medicine | 1715 | 47.0 | 44.4 | 1568 | 147 | 51.9 | 23.6 | <0.001 | 50.0 | 22.0 | <0.001 | 1616 | 99 | 50.9 | 21.1 | <0.001 | 48.8 | 20.2 | <0.001 |
| 3-4 medicine | 1034 | 28.4 | 28.7 | 849 | 185 | 28.1 | 29.7 |  | 28.6 | 29.3 |  | 905 | 129 | 28.5 | 27.5 |  | 29.0 | 27.0 |  |
| >= 5 medicine (polypharmacy) | 896 | 24.6 | 26.9 | 605 | 291 | 20.0 | 46.7 |  | 21.4 | 48.8 |  | 655 | 241 | 20.6 | 51.4 |  | 22.2 | 52.7 |  |
| On supplements only | 1021 | 19.1 | 18.2 | 864 | 157 | 18.4 | 23.5 | 0.002 | 17.4 | 22.9 | 0.001 | 900 | 121 | 18.5 | 24.4 | 0.001 | 17.5 | 24.2 | <0.001 |
| **Types of medicines** |  |  |  |  |  |  |  |  |  |  |  |  |  |  |  |  |  |  |  |
| On any Statin | 1643 | 30.5 | 31.1 | 1325 | 318 | 28.1 | 46.9 | <0.001 | 28.4 | 47.1 | <0.001 | 1395 | 248 | 28.6 | 49.0 | <0.001 | 28.8 | 49.2 | <0.001 |
| On ACE or ARB | 1279 | 23.7 | 25.3 | 961 | 318 | 20.4 | 46.9 | <0.001 | 21.5 | 47.2 | <0.001 | 1028 | 251 | 21.1 | 49.6 | <0.001 | 22.2 | 49.4 | <0.001 |
| On antihypertensive drugs | 780 | 14.6 | 15.7 | 548 | 232 | 11.7 | 34.8 | <0.001 | 12.4 | 34.9 | <0.001 | 592 | 188 | 12.2 | 37.9 | <0.001 | 12.9 | 37.37 | <0.001 |
| On any ACE inhibitors | 681 | 12.6 | 13.9 | 512 | 169 | 10.9 | 24.9 | <0.001 | 11.9 | 25.3 | <0.001 | 548 | 133 | 11.1 | 26.3 | <0.001 | 12.3 | 26.5 | <0.001 |
| On ARB | 620 | 11.5 | 11.8 | 463 | 157 | 9.8 | 23.2 | <0.001 | 9.9 | 23.0 | <0.001 | 495 | 125 | 10.1 | 24.7 | <0.001 | 10.3 | 24.3 | <0.001 |
| On any NSAID | 340 | 6.3 | 6.6 | 279 | 61 | 5.9 | 9.0 | 0.002 | 6.1 | 9.3 | 0.004 | 292 | 48 | 5.9 | 9.5 | 0.002 | 6.1 | 9.8 | 0.003 |
| On any blood glucose lowering drug – excl. insulin | 273 | 5.1 | 5.5 | 200 | 73 | 4.3 | 10.8 | <0.001 | 4.6 | 10.6 | <0.001 | 210 | 63 | 4.3 | 12.4 | <0.001 | 4.7 | 11.9 | <0.001 |
| *ARB = Angiotensin II Antagonist, ACE = Angiotensin- converting enzymes, NSAID= non-steroidal anti-inflammatory drugs.*  *UW- unweighted, W- weighted, ^b^race free, P- value calculated applying chi-square test, P < 0.05 indicates statistically significant.* | | | | | | | | | | | | | | | | | | | |

**Supplementary Table 4: Comparing distribution of medication among CVD and diabetic with CKD patients, based on CKD-EPI 2012_(Scr-CysC)_- Weighted**

| **Medication** | **Total (N=5386)**  **n (%)** | **CVD** | | | **Diabetes** | | | **CKD** | | |
| --- | --- | --- | --- | --- | --- | --- | --- | --- | --- | --- |
|  |  | **No (N=4822)**  **n (%)** | **Yes (N=564)**  **n (%)** | **P-value** | **No(N=4893)**  **n (%)** | **Yes (N=442)**  **n (%)** | **P-value** | **No(N=4708)**  **n (%)** | **Yes (N=678)**  **n (%)** | **P-value** |
| No meds | 1741 (31.2) | 1722(34.8) | 19 (2.7) | <0.001 | 1693 (33.5) | 29 (6.0) | <0.001 | 1686 (35.2) | 55 (7.5) | <0.001 |
| 1-2 meds | 1715 (30.6) | 1666 (33.5) | 49 (8.9) |  | 1632 (32.2) | 62 (13.4) |  | 1568 (32.4) | 147 (20.3) |  |
| 3-4 meds | 1034 (19.7) | 859 (18.5) | 175 (29.2) |  | 926 (19.5) | 102 (23.6) |  | 849 (18.5) | 185 (27.1) |  |
| >=5meds (polypharmacy) | 896 (18.5) | 575 (13.2) | 321 (59.2) |  | 642 (14.8) | 249 (57.0) |  | 605 (13.9) | 291(45.1) |  |

**Supplementary Table 5: Univariate and multivariate logistic regression models and their comparison, based on CKD-EPI 2021_(Scr-CysC)_** **(weighted)**

| **Characteristics** | **Univariate logistic regression** | | **Multivariate logistic regression** | | | | | |
| --- | --- | --- | --- | --- | --- | --- | --- | --- |
|  |  |  | **Model 1 (N= 5386)** | | **Model 2 (N=5321)** | | **Model 3 (N= 5307)** | |
|  | **OR [95%CI]** | **P-value** | **AOR [95%CI]** | **P-value** | **AOR [95%CI]** | **P-value** | **AOR [95%CI]** | **P-value** |
| Age | 1.18 [1.16 - 1.20] | <0.001 | 1.16[1.14 - 1.18] | <0.001 | 1.15 [1.13 - 1.17] | <0.001 | 1.15 [1.12 - 1.17] | <0.001 |
| **Sex** |  |  |  |  |  |  |  |  |
| Men | 1 [-] |  | 1 [-] |  | 1 [-] |  | 1 [-] |  |
| Women | 1.45 [1.20 - 1.74] | <0.001 | 1.09 [0.88 - 1.38] | 0.415 | 1.28 [1.01 - 1.64] | 0.045 | 1.28[1.01 - 1.63] | 0.045 |
| **Education** | | |  |  |  |  |  |  |
| Tertiary | 1 [-] |  | 1 [-] |  | 1 [-] |  | 1 [-] |  |
| Primary | 4.50 [ 3.42 - 5.93] | <0.001 | 1.82 [1.36 - 2.44] | <0.001 | 1.56 [1.17 - 2.11] | 0.002 | 1.44 [1.07 - 1.94] | 0.016 |
| Secondary | 1.68 [1.28 - 2.21] | <0.001 | 1.57 [1.17 - 2.11] | 0.028 | 1.45 [1.07 - 1.96] | 0.015 | 1.36 [1.01 - 1.85] | 0.044 |
| **Employment** | |  |  |  |  |  |  |  |
| Employed | 1 [-] |  | 1 [-] |  | 1 [-] |  | 1 [-] |  |
| Retired | 11.58 [8.12 -15.16.51] | <0.001 | 1.80 [1.21 - 2.68] | 0.003 | 1.69 [1.12 - 2.56] | 0.013* | 1.57 [1.04 - 2.39] | 0.032 |
| Unemployed | 6.81[4.64 - 10.0] | <0.001 | 2.29 [1.53 - 3.44] | <0.001 | 2.09 [1.39 - 3.17] | <0.001* | 1.93 [1.27 - 2.93] | 0.002 |
| **CVD** |  |  |  |  |  |  |  |  |
| No | 1 [-] |  |  |  | 1 [-] |  | 1 [-] |  |
| Yes | 4.03 [3.19 - 5.10] | <0.001 |  |  | 1.66 [1.25 - 2.19] | <0.001* | 1.57 [1.18 - 2.09] | 0.001 |
| **Diabetes** |  |  |  |  |  |  |  |  |
| No | 1 [-] |  |  |  | 1 [-] |  | 1 [-] |  |
| Yes | 2.89 [2.20 - 3.78] | <0.001 |  |  | 1.76 [1.27 - 2.44] | <0.001* | 1.69 [1.22 - 2.36] | 0.001 |
| **Hypertension** |  |  |  |  |  |  |  |  |
| No | 1 [-] |  |  |  | 1 [-] |  | 1 [-] |  |
| Yes | 5.34 [3.90 - 7.32] | <0.001 |  |  | 1.82 [1.31 - 2.54] | <0.001* | 1.84 [1.32 - 2.56] | <0.001 |
| **COPD** |  |  |  |  |  |  |  |  |
| No | 1 [-] |  |  |  | 1 [-] |  | 1 [-] |  |
| Yes | 1.37 [1.05 - 1.78] | 0.021 |  |  | 1.17 [0.86 - 1.59] | 0.324 | 1.10 [0.80 - 1.51] | 0.532 |
| **Cancer** |  |  |  |  |  |  |  |  |
| No | 1 [-] |  |  |  | 1 [-] |  | 1 [-] |  |
| Yes | 2.23 [1.56 - 3.18] | <0.001 |  |  | 1.68 [1.14 - 2.48] | 0.009 | 1.63 [1.10 - 2.40] | 0.014 |
| **BMI** |  |  |  |  |  |  |  |  |
| Normal | 1 [-] |  |  |  | 1 [-] |  | 1 [-] |  |
| Overweight | 1.01 [0.75 - 1.36] | 0.954 |  |  | 1.07 [0.76 - 1.50] | 0.696 | 1.08 [0.77 - 1.51] | 0.664 |
| Obesity | 1.94 [1.46 - 2.58] | <0.001 |  |  | 2.11 [1.52 - 2.93] | <0.001 | 2.09 [1.50 - 2.89] | <0.001 |
| **Hospital admission (within the past 12 months** | |  |  |  |  |  |  |  |
| No | 1 [-] |  |  |  |  |  | 1 [-] |  |
| Yes | 2.22 [1.75 - 2.83] | <0.001 |  |  |  |  | 1.55 [1.13 - 2.11] | 0.006 |
| **Have medical/GP Card** |  |  |  |  |  |  |  |  |
| No | 1 [-] |  |  |  |  |  | 1 [-] |  |
| Yes | 8.32 [6.56 - 10.56] | <0.001 |  |  |  |  | 1.38 [1.05 - 1.81] | 0.021 |
| **AUC-ROC** |  |  | 0.85 | | 0.86 | | 0.87 | |

*All the presented results are weighted, Chronic Obstructive Pulmonary Disease (COPD) included chronic bronchitis, emphysema, asthma. AOR Adjusted Odds Ratio, BMI (Body mass Index): Normal (18.5 - 24.9kg/m)2, Overweight (BMI 25 –29.9 kg/m2), Obesity (BMI >= 30kg/m2, *Statistically Significant at p < 0.05.*

**Supplementary Table 6: eGFR distribution(prevalence) by sex distribution of participants, based on CKD-EPI 2012** _(Scr-CysC)_ **and CKD-EPI 2021** _(Scr-CysC)_

|  | **Total (N)** | **eGFR <30 ml/min** | | | **eGFR 30-44 ml/min** | | | **eGFR 45-59 ml/min** | | | **eGFR 60-89 ml/min** | | | **eGFR >=90 ml/min** | | |
| --- | --- | --- | --- | --- | --- | --- | --- | --- | --- | --- | --- | --- | --- | --- | --- | --- |
|  |  | **Cases (n)** | **Prevalence** | | **Cases (n)** | **Prevalence** | | **Cases (n)** | **Prevalence** | | **Cases (n)** | **Prevalence** | | **Cases (n)** | **Prevalence** | |
|  |  |  | **UW (%)** | **W (%)** |  | **UW (%)** | **W (%)** |  | **UW (%)** | **W (%)** |  | **UW (%)** | **W (%)** |  | **UW (%)** | **W (%)** |
| **CKD-EPI 2012**_(Scr-CysC)_ | |  |  |  |  |  |  |  |  |  |  |  |  |  |  |  |
| **Overall** | **5386** | **45** | **0.8** | **1.1** | **179** | **3.3** | **4.2** | **454** | **8.4** | **9.5** | **3071** | **57.0** | **55.9** | **1637** | **30.4** | **29.4** |
| **Sex** |  |  |  |  |  |  |  |  |  |  |  |  |  |  |  |  |
| Men | 2504 | 21 | 0.8 | 0.8 | 85 | 3.4 | 3.8 | 172 | 6.9 | 6.9 | 1439 | 57.4 | 56.2 | 788 | 31.5 | 32.3 |
| Women | 2881 | 24 | 0.8 | 1.4 | 94 | 3.3 | 4.5 | 282 | 9.8 | 11.9 | 1632 | 56.6 | 55.7 | 849 | 29.5 | 26.5 |
| **CKD-EPI 2021**_(Scr-CysC)_ **^b^** | | | |  |  |  |  |  |  |  |  |  |  |  |  |  |
| **Overall** | **5386** | **37** | **0.7** | **0.9** | **136** | **2.5** | **3.3** | **333** | **6.2** | **7.1** | **2635** | **48.9** | **48.6** | **2245** | **41.7** | **40.1** |
| **Sex** |  |  |  |  |  |  |  |  |  |  |  |  |  |  |  |  |
| Men | 2505 | 17 | 0.7 | 0.7 | 68 | 2.7 | 3.2 | 135 | 5.4 | 5.5 | 1215 | 48.5 | 47.1 | 1070 | 42.7 | 43.5 |
| Women | 2881 | 20 | 0.7 | 1.1 | 68 | 2.4 | 3.4 | 198 | 6.9 | 8.6 | 1420 | 49.3 | 50.1 | 1175 | 40.8 | 36.8 |
| *UW- unweighted, W- weighted.* **^b^** *race free* | | | | | | | | | | | | | | | | |
